# Facial emotion recognition in children of parents with a mental illness

**DOI:** 10.3389/fpsyt.2024.1366005

**Published:** 2024-06-13

**Authors:** Naomi Leona Werkmann, Arleta Angelika Luczejko, Klara Hagelweide, Rudolf Stark, Sarah Weigelt, Hanna Christiansen, Meinhard Kieser, Kathleen Otto, Corinna Reck, Ricarda Steinmayr, Linda Wirthwein, Anna-Lena Zietlow, Christina Schwenck, the COMPARE-family research group

**Affiliations:** ^1^ Department of Clinical Child and Adolescent Psychology, Justus Liebig University Giessen, Giessen, Germany; ^2^ Department of Rehabilitation Sciences, Technical University Dortmund, Dortmund, Germany; ^3^ Department of Psychotherapy and Systems Neuroscience, Justus-Liebig University Giessen, Giessen, Germany; ^4^ Department of Psychology, Clinical Child and Adolescent Psychology, Philipps University Marburg, Marburg, Germany; ^5^ Institute of Medical Biometry, University of Heidelberg, Heidelberg, Germany; ^6^ Department of Work and Organizational Psychology, Philipps-University Marburg, Marburg, Germany; ^7^ Department of Psychology, Ludwig-Maximilians-Universität München, Munich, Germany; ^8^ Department of Psychology, Technical University Dortmund, Dortmund, Germany; ^9^ Clinical Child and Adolescent Psychology, Department of Psychology, Technische Universität Dresden, Dresden, Germany

**Keywords:** transgenerational transmission of mental disorders, facial emotion recognition, parents with mental illness, children of parents with mental illness (COPMI), multimodal assessment

## Abstract

**Objective:**

Facial emotion recognition (FER) is a fundamental social skill essential for adaptive social behaviors, emotional development, and overall well-being. FER impairments have been linked to various mental disorders, making it a critical transdiagnostic mechanism influencing the development and trajectory of mental disorders. FER has also been found to play a role in the transgenerational transmission of mental disorders, with the majority of research suggesting FER impairments in children of parents with a mental illness (COPMI). Previous research primarily concentrated on COPMI of parents with internalizing disorders, which does not cover the full spectrum of outpatient mental health service populations. Furthermore, research focuses on varying components of FER by using different assessment paradigms, making it challenging to compare study results. To address these gaps, we comprehensively investigated FER abilities in COPMI using multiple tasks varying in task characteristics.

**Methods:**

We included 189 children, 77 COPMI and 112 children of parents without a diagnosed mental illness (COPWMI), aged 6 to 16 years. We assessed FER using three tasks with varying task demands: an emotional Go/NoGo task, a morphing task, and a task presenting short video sequences depicting different emotions. We fitted separate two-level hierarchical Bayesian models (to account for sibling pairs in our sample) for reaction times and accuracy rates for each task. Good model fit was assured by comparing models using varying priors.

**Results:**

Contrary to our expectations, our results revealed no general FER deficit in COPMI compared to COPWMI. The Bayesian models fitted for accuracy in the morphing task and Go/NoGo task yielded small yet significant effects. However, Bayes factors fitted for the models suggested that these effects could be due to random variations or noise in the data.

**Conclusions:**

Our study does not support FER impairments as a general feature of COPMI. Instead, individual factors, such as the type of parental disorder and the timing of its onset, may play a crucial role in influencing FER development. Future research should consider these factors, taking into account the diverse landscape of parental mental disorders.

## 1 Background

### 1.1 Facial emotion recognition

Facial emotion recognition (FER) describes the ability to correctly decipher emotional expressions in human faces, which is crucial for adaptive social behaviors, emotional development, and overall well-being (1). This fundamental social skill emerges early in life, with the first precursors presenting in infancy (2–5). FER continues to improve throughout childhood (6) and reaches peak performance in early adulthood before declining again (7). Research suggests that genetic and environmental factors play a role in the development of FER (8–11). Parents are a critical factor in FER development, especially during early childhood, because they shape their children’s emotional environment in direct ways, such as explicit teaching of emotions, as well as indirectly through their own beliefs about emotions and their own FER abilities (12).

Impaired FER is linked to various mental disorders in both children and adults (13), such as internalizing disorders (14–16), schizophrenia (17), borderline personality disorder (18), and externalizing disorders (19). It is therefore considered to be an important transdiagnostic feature relevant to the development and course of mental illness (20). FER also serves as a foundational element for further emotion processing, regulation, empathy, and, consequently, proficient social interaction (9)—each of which is compromised across a spectrum of disorders as well (20). Beyond being a transdiagnostic feature in mental illness, research shows that abnormal FER predates the onset and development of mental disorders (9, 21–23).

### 1.2 FER assessment

FER research has gained popularity over the past years, with assessment methods becoming more diverse. While early research on FER focused solely on identifying emotions in static pictures, more recent studies use a broader variety of assessment paradigms. This evolution is in part due to improvements in assessment technologies but also theoretically based as emotional expressions in natural contexts are often swift and subtle, meaning static pictures are not as ecologically valid as other stimulus types (24, 25). However, as the landscape of studies on FER becomes more diverse regarding study design and employed tasks, the results also become more challenging to compare as task characteristics have a significant influence on study outcomes (26, 27). One important factor to consider is the outcome measure used to assess FER. Some studies focus on accuracy rates, while others focus on reaction speed or sensitivity in FER assessment. These measures likely represent distinct components of FER and rely on different cognitive processes (26–28). Differences in the outcome measures also come with different practical implications: Because facial expressions change rapidly and are often subtle in realistic contexts, results measuring sensitivity toward emotional expressions might be more relevant than accuracy rates when deriving clinical implications from research findings. Furthermore, factors such as stimulus differences (e.g., ethnicity or sex of the faces), contextual clues, and response requirements can influence study outcomes (27). In addition, the answer formats differ across studies. While in some studies participants are asked to name the presented emotion without any prompt, other studies rely on forced-choice formats. Another critical difference between studies is whether the stimuli are static or contain motion. Because emotional expressions in human faces are dynamic, it can be argued that results derived from static stimuli display lower ecological validity than results gained with dynamic stimuli.

Taken together, FER is a relevant feature in the context of mental disorders and has gained popularity as a research subject in recent years. However, the diversity in study design and task characteristics must be considered when interpreting and comparing study outcomes.

### 1.3 FER in children of parents with a mental illness

Children of parents with a mental illness (COPMI) exhibit higher subclinical internalizing and externalizing symptom rates (29), compared to children of parents without a mental illness (COPWMI), and have a significantly elevated risk of mental disorder development (20, 30, 31). Considering parents’ vital role in shaping their children’s emotional environment in various ways (12) in conjunction with the significance of FER in the context of mental illness, it is plausible to assume FER impairments in COPMI.

However, research on FER in COPMI yields heterogeneous results. While the majority of studies suggest FER *impairments* in COPMI to varying extents, a few studies found FER *improvements* for specific emotions compared to COPMWI. Research on FER in children of parents with depression provides evidence supporting the idea of FER deficits (21, 32). Joormann et al. (32) assessed FER in daughters of mothers with a history of depression using facial stimuli morphing from a neutral to an emotional facial expression (*morphing task*). They found daughters of mothers with a history of depression to make more errors in identifying anger and to require higher intensity to identify sad facial expressions accurately. Mannie et al. (21) add to this body of evidence. In their study, young adults who had a biological parent with a history of depression performed significantly slower in an emotional categorization task. Furthermore, research found that FER deficits among COPMI were discernible from an early age (33–35). In a study by Székely et al. (33), depressive symptoms at any time point during the child’s life significantly predicted impaired accuracy in an emotion identification task using static pictures in preschoolers. Meiser et al. (34) found aligning evidence, stating that preschool-aged children whose mothers had suffered from postpartum depression or anxiety disorders performed significantly worse on emotion labeling tasks. Also, FER impairments do not seem to be specific to children of parents with depression, because research found FER impairments in children of parents with other disorders as well (6, 24, 36–41). Hanford et al. (37) assessed FER in children of parents diagnosed with bipolar disorder using a task presenting emotional expressions in static faces at different intensities. They included symptomatic as well as asymptomatic teenage children in their study. Here, children of parents diagnosed with bipolar disorder made more errors across emotions regardless of their own diagnostic status. Sharma et al. (38) add to these findings because they found unaffected school-aged children of parents diagnosed with bipolar disorder to conduct more errors in overall emotion recognition and specifically in the recognition of fear on a static picture task. In a study by Bilodeau et al. (6), the existing evidence was expanded to children of parents with panic disorder. They assessed FER in unaffected children of parents with a current or past history of panic disorder and found those children made more errors in recognizing fear and anger as well as sadness. Horton et al. (40) noted that children of parents with diagnosed schizophrenia show lower accuracy as well as recognition speed across all emotions and for fear specifically.

However, as mentioned above, a few studies yielded contrasting results. Lopez-Duran et al. (42) assessed FER in children whose parents have a documented history of childhood-onset depression via a morphing task. They found that boys, but not girls, of parents with a history of depression require lower intensity levels to correctly identify sadness in a morphing task. Burkhouse et al. (43) assessed FER in children of mothers with a history of depression using a forced-choice emotion identification task. They found that children of mothers with a history of depression exhibit increased sensitivity in detecting sad faces and reduced sensitivity in detecting happy faces only if the children carried a specific allele in their oxytocin receptor gene. Children who did *not* have this genetic predisposition did not show any differences in FER compared to the control group. Joormann et al. (44) added to this body of evidence because they found that daughters of mothers with depression display selective attention toward negative facial expressions, while daughters of healthy mothers exhibited selective attention to positive facial expressions after a negative mood induction paradigm. The “stress acceleration hypothesis” explains these results. It proposes FER *improvements* for specific emotions in COPMI as an adaptive survival strategy to a negative environment (45). According to this theory, it can be beneficial for children of parents with depression to be sensitive toward sad facial expressions, because they are a relevant social cue in their home environment and may be connected to certain expectations regarding the child’s behavior. However, the studies mentioned above only show FER improvements for specific emotions and in specific contexts (e.g., children with certain genetic predispositions), suggesting that FER *improvements* are not a general feature related to parental mental illness.

Taken together, while a few studies find FER improvements for specific emotions and/or in specific contexts (42–45), most research suggests FER impairments in COPMI (6, 8, 10, 11, 21, 32–34, 36–41). However, due to differences in study designs and employed tasks, it remains unclear whether these deficits are specific to certain emotions, tasks, or stimuli. Furthermore, almost all research on FER in COPMI focuses on children of parents with *internalizing* disorders. However, this is not representative of populations in the mental healthcare system. To expand existing findings and to ensure generalizability for populations assessing outpatient mental health services, it is essential to assess a more heterogeneous group of COPMI regarding parental disorder type.

Thus, our study employs different tasks with varying task demands in a COPMI sample representative for populations assessing outpatient mental health services to get deeper insights into FER in COPMI. In line with the majority of research, we hypothesized that COPMI would show impairments in FER across all three tasks. Specifically, we hypothesized that COPMI would show lower accuracy rates and higher reaction times in an emotional Go/NoGo task (H1). Second, we expected that COPMI would show lower accuracy rates and higher reaction times in a morphing task (H2). Third, we expected lower accuracy rates in correctly naming emotions in a non-speeded task presenting emotional video sequences (H3).

## 2 Method

### 2.1 Study design

The present study was conducted in a cross-sectional setting. Participants took part in a questionnaire assessment as well as a lab assessment. See Section 2.5. for more details. The present study is part of a randomized controlled multicenter study of a preventive intervention for COPMI in Germany (COMPARE-family) and its add-on project COMPARE-emotion). The projects are described in detail in the study protocols (46, 47). The local ethics committee approved the study, and informed consent was given by each parent and child participating in COMPARE-family and COMPARE-emotion.

### 2.2 Eligibility criteria

The inclusion criteria were as follows: a) children between 6 and 16 years of age for COPMI, b) a parent with a mental illness according to the Diagnostic and Statistical Manual of Mental Disorders (DSM-5) (48) assessed via a semistructured clinical interview and for COPWMI, and c) parents without a current or past mental disorder and without current or past psychotherapeutic treatment. The exclusion criteria were a) insufficient German language skills of children and parents; b) for COPMI, severe impairment of the children requiring comprehensive treatment; c) a parent ongoing outpatient or inpatient treatment or continuous use of benzodiazepines; and d) for COPWMI, a *T*_CBCL_ ≥62.

### 2.3 Recruitment

COPMI were recruited as part of the COMPARE-family project (46, 47). Parents with a mental illness were primarily recruited through university outpatient clinics in Gießen and Bochum in Germany. In Gießen, families were additionally contacted via letters to families with children in the corresponding age range provided by the local registry office, public advertisement, information material in inpatient psychiatric clinics and psychotherapeutic practices, and the University’s internal mailing list. COPWMI were recruited in Gießen and Dortmund as part of the add-on project COMPARE-emotion via the respective research group’s database of former study participants, mailings of families with children in the corresponding age range, social media advertisement, and public advertisement in schools and daycare centers.

### 2.4 Measures

#### 2.4.1 Diagnostic measures

##### 2.4.1.1 Socioeconomic status (SES)

SES was assessed according to the Studie zur Gesundheit von Kindern und Jugendlichen in Deutschland (KiGGS) study (49). The SES index ranges between 3.0 and 21.0 and considers education, professional qualifications, status, and net household income. The index can be used as a metric variable or grouped into low, middle, and high SES (50). We assessed SES in a metric way.

##### 2.4.1.2 Psychopathology of children

We used the German parent-report version of the *Child Behaviour Checklist 6–18R* (*CBCL 6–18R* (51);) to assess the psychopathology of COPMI and COPWMI. Items are aggregated into three superordinate scales (externalizing problems, internalizing problems, and total problems). Good to very good internal consistencies were reported for the CBCL 6–18R (Cronbach’s alpha =.82 –.93 (51);). We derived *T*-values for analyses according to the norm tables provided in the questionnaire manual.

##### 2.4.1.3 Parental psychopathology

We used the German version of the self-reported questionnaire *Brief Symptom Inventory* (*BSI* (52);) to assess parents’ psychopathology. The BSI contains 53 items rated on a five-point Likert scale from 0 (*not at all*) to 4 (*very much*). We aggregated items to the Global Severity Index (GSI). DerogatisKlicken oder tippen Sie hier, um Text einzugeben (52). reported very good internal consistency of GSI (Cronbach’s alpha = .97). Parents of COPWMI were excluded if GSI was above the cutoff value (*T*_GSI_ ≥ 62). For further analyses, we derived *T*-values according to the norm tables provided in the questionnaire manual.

##### 2.4.1.4 Diagnostic interview for mental disorders (DIPS)

The DIPS (53) is a semistructured diagnostic interview to determine mental disorders according to the DSM-5 (48). Trained clinicians conducted it to assess the diagnostic status of the parents of COPMI. Previous studies report high interrater reliability (.72 < *κ* <.92) and test–retest reliabilities, mostly in the range of.62 to.94 (53).

#### 2.4.2 Experimental measures

##### 2.4.2.1 Emotional Go/NoGo task

This task was developed by Tottenham et al. (54) as a means to assess different components of emotion processing in children. The stimuli for this task consisted of grayscale pictures displaying neutral, sad, and angry faces, derived from the NimStim set (55), including five male and five female faces of White, Black, and Asian individuals, depicting sad, fearful, or neutral expressions. The experiment consisted of six blocks with 48 trials each. Seventy-three percent (35 trials) were Go trials, and 27% (13 trials) were NoGo trials. In each block, one expression (neutral, fearful, sad) was the Go stimulus and another expression was the NoGo stimulus. The order of the blocks was randomized for each participant, while the stimulus order within a block was kept consistent. The stimuli were presented for 1,000 ms with 2,000 ms between stimuli. In between stimuli, a white fixation cross was presented. The children were instructed to press the space bar for each Go stimulus and not to react to NoGo stimuli as fast and with as few mistakes as possible. Reactions were only counted within the stimulus presentation interval of 1,000 ms. In line with the suggestions by Tottenham et al. (54), we calculated *D-prime* as a measure of accuracy by subtracting z-standardized false alarms (reactions to NoGo stimuli) from z-standardized correct answers (reactions to Go stimuli) for each participant. We also computed mean reaction times for the Go stimuli for each participant across all blocks as a measure for FER speed, excluding reaction times lower than 100 ms.

##### 2.4.2.2 Morphing task

Morphing tasks are well-established tasks to assess emotion recognition using dynamic facial stimuli. The morphing task included in our study was developed in reference to a morphing task by Schwenck et al. (56). Children watched 48 film clips of 9 s length that started with a neutral facial expression and changed continuously to an emotional expression. The stimuli consisted of 30 different White faces, 15 male and 15 female faces, displaying the emotions anger, sadness, joy, and fear. The faces were sourced from the KDEF database (57). Participants were instructed to press the spacebar on the computer keyboard as soon as they recognized the person’s emotion and to name it out loud to the experimenter afterward. If participants did not press the spacebar during the 9-s-long video, they were encouraged to name or guess the presented emotion. If the children did not name any emotion, the four possible answers were presented on the screen, and children were asked which of those emotions they saw. Experimenters entered the emotion via keystroke. Reaction times of correct trials and accuracy rates were assessed. In a control task, shapes morphed into animals, and children were asked to press the space bar as soon as they recognized the animal and then name the animal they recognized. Reaction times and accuracy rates from this control task were included as covariates in further analyses to ensure possible impairments are specific to identifying emotions.

##### 2.4.2.3 Task presenting emotional video sequences

This task was *ad-hoc* developed and tested in a pilot study in order to assess emotion recognition as well as compassion, mimicry, and arousal in children while watching emotional video clips. The participants were presented with eight short videos (between 23 and 36 s). Each clip featured a protagonist (a child or teenager) displaying either fear, sadness, happiness, or anger. The video clips were derived from the German children’s TV program. After each clip, participants were asked to a) name the emotion displayed, b) rate their own arousal while watching the video between 1 to 5, and c) rate their compassion for the protagonist on a scale of 1 to 5. After each clip, a marine life clip (approx. 30 s) was presented to avoid carryover effects for the physiological data assessed during the task. When asked to name the presented emotion, participants were aware that the emotion had to be either fear, sadness, happiness, or anger. Participants were given an unlimited amount of time to answer. Accuracy rates were calculated as percentages of correct answers across all trials.

### 2.5 Procedure

All participants and their parents gave written informed consent before the assessment. Parents received an expense allowance of €50 (COPWMI)/€15 (COPMI). Parents of COPMI were additionally included in the longitudinal intervention study (COMPARE-family) and, therefore, received disorder-specific evidence-based cognitive behavioral treatment after this assessment. Questionnaires were completed by parents online prior to or during the in-person assessment. During the in-person assessment, children were asked to complete three computerized tasks and fill in questionnaires, while the adjoining parent was interviewed by a trained professional regarding their children’s diagnostic status. In a separate appointment, the parents of COPMI completed a clinical interview regarding their own diagnostic status.

Children completed the Go/NoGo task (54), morphing task (56), and a task presenting emotional video clips developed specifically for our study in randomized order. For all computer tasks, children were positioned in a quiet room, in 60 cm distance from a stationary computer screen (24 in., 1,920 × 1,080 pixels). The tasks were presented via the *E-Prime* software (58).

### 2.6 Analysis strategy

We performed statistical analyses using *SPSS* version 28.0 (59) and *RStudio* version 2023.06.2 (60) along with additional packages *brms* (61) and *bayestestR* (62). First, mean reaction times were computed as the mean of all reaction times across a correct trial. Accuracy rates were computed as the number of correct trials divided by the number of total trials. To address the need for confounding variables in further analyses, we examined possible differences in demographic characteristics between COPMI and COPWMI (see **Table 1**). In all analyses, age, sex, and SES were included as covariates. Two-level Bayesian hierarchical linear models with group (COPMI vs. COPWMI) as fixed and family (to account for siblings) as random effects were fit using the *brms* package (61) to test our hypotheses. Models with a normal skew distribution were chosen for all outcome variables, except for reaction times in the morphing task, to account for skewness in the data distribution (63). For each model, 95% credibility intervals (CIs) were calculated, and statistical significance for regression coefficients was identified when the CI did not include zero. We fit separate models for mean accuracy and reaction times in the Go/NoGo task and the morphing task, as well as accuracy in the task presenting emotional video sequences. Models yielding significant effects were split up into single models for each emotion to be able to identify the cause of the effect. Bayes factor (BF) was calculated for each model using the Savage–Dickey ratio via the *bayestestR* package (62) as a means to assess the relative evidence for the alternative over the null model (64). BF smaller than 1.00 suggests that evidence favors the null hypothesis, while BF greater than 1.00 suggests that evidence favors the postulated alternative hypothesis. Priors in each model were adjusted to account for beliefs about general data distributions as well as effect sizes to ensure the best model fit, taking RMSEA, BIC, bulk-ESS, and tail-ESS into account. To ensure that our sample was large enough to detect possible FER differences, we took several parameters, such as R-hat values, effective sample sizes (bulk- and tail-ESS), and BIC into account. Bayesian methods do not rely on power analysis in the classical/frequentist sense to determine the statistical power/required sample size, but rather the model fit determines whether the results are reliable. Generally, Bayesian models are well equipped to model data even in small samples, as they do not rely on asymptotics (65). However, in small samples, the models become more sensitive to the employed priors (66). To address this prior sensitivity, we compared models with varying priors and compared BIC as well as bulk- and tail-ESS. So, while sample size does have an effect on the Bayes models, we assured that our sample was large enough by ensuring the model fit parameters were very good.

**Table 1:** Demographic characteristics of the participants and means and standard deviations of psychopathological symptoms of children and parents.

	COPMI (*N* = 77)	COPWMI (*N* = 112)	*p*
	*M* (SD)/*N* (%)	*M* (SD)/*N* (%)	
Children			
Age	10.23 (2.78)	10.86 (2.66)	.122
Sex (female)	44 (48.35)	56 (57.14)	.836
CBCLext (*T*-score)	49.86 (9.71)	44.58 (8.36)	<.001
CBCLint (*T*-score)	55.95 (10.24)	46.64 (10.32)	<.001
2nd child	20	32	
3rd child	–	4	
Parents			
Age	41.91 (6.05)	43.55 (5.72)	.088
Sex (female)	54 (81.80)	74 (85.10)	.594
SES	14.27 (3.35)	18.24 (2.27)	<.001
BSI GSI (*T*-score)	62.68 (13.54)	39.84 (9.33)	<.001

CBCLext, Externalizing Subscale Score of the Child Behavior Checklist; CBCLint, Internalizing Subscale Score of the Child Behavior Checklist; SES, socioeconomic status; BSI GSI, Global Severity Index of the Brief Symptom Inventory.

## 3 Results

### 3.1 Participants

In total, 189 children were included in this study, with 77 COPMI and 112 COPWMI. Children ranged in age from 6 to 16 years (*M* = 10.60, SD = 2.27), and 50.30% (*n* = 95) were female children. In the COPMI group, 20 children were siblings; in the COPWMI group, 36 children were siblings. For more detailed demographic characteristics, see **Table 1**. COPMI and COPWMI did not differ significantly in age and gender or parents’ age and gender. There were significant group differences between the groups in children’s psychopathological symptoms and socioeconomic status (SES; see **Table 1**). In both groups, most of the parents were mothers (81.80% in the COPMI group and 85.10% in the COPWMI group), but there was no significant difference in gender distribution between the groups (see **Table 1**). The majority of parental primary diagnoses were depressive disorders (48.1%). On average, parents had *M* = 1.83 diagnoses (SD = 1.17, range from 1 to 5), and the severity of the primary diagnosis ranged from 4 to 8 according to the DIPS (53) (*M* = 5.89, SD = .75; ranged between 0 and 8, diagnosis being clinically relevant from 4 and above). For further classifications of primary diagnoses, see **Table 2**.

**Table 2:** Classification of primary diagnoses in parents with mental illness.

	*N*	%
Depressive disorders	37	48.1
Anxiety disorders	12	15.6
Trauma- and stressor-related disorders	21	27.3
Somatic symptom and related disorders	2	3.6
Feeding and eating disorders	2	3.6
Schizophrenia spectrum and other psychotic disorders	3	3.9

### 3.2 Hypothesis testing

Means and standard deviations for each outcome measure are reported separately for both groups (COPMI vs. COPWMI) in **Table 3**. Bayesian correlational measures between the covariates and the outcome measures can be found in the **Supplementary Material**. Because in Bayesian modeling, decisions regarding the inclusion of covariates in further analyses are not solely based on correlation measures or coefficients but rather on a holistic consideration of the research question, prior knowledge, and the underlying mechanisms, SES, gender, and age were included in the Bayes models. The model fit for each model indicated that Bayes models including all three variables were the best fit for our data.

**Table 3:** Means and standard deviations for each task separately for COPMI and COPWMI as well as Bayesian analysis statistics for each outcome variable.

	Mean (SD)		Standardized estimate	SD	95% CI_lower_	95% CI_upper_	Bayes factor
	COPMI	COPWMI					
**Accuracy Go/NoGo task (D-prime)**	**−.188 (1.707)**	**.110 (1.505)**	**−.197**	**.489**	**−.293**	**−.101**	**.839**
RT Go/NoGo task	340.772 (84.754)	360.437 (54.382)	−3.876	9.005	−21.293	14.064	.027
Accuracy MT baseline	.868 (1.32)	.900 (.098)					
RT MT baseline	7.493 (.749)	7.255 (.733)					
**Accuracy MT**	**.906 (.069)**	**.873 (.088)**	**−.024**	**.011**	**−.045**	**−.003**	**.187**
RT MT	5.790 (.807)	5.535 (.853)	.095	.165	−.228	.417	<.001
Accuracy VST	.952 (.085)	.956 (.119)	.001	.006	−.011	.014	.008

Significant effects are printed in bold.

MT, morphing task; RT, reaction times; VST, task depicting emotional video sequences; CI, confidence interval; SES, socioeconomic status.

#### 3.2.1 FER differences in the emotional Go/NoGo task

We calculated D-prime per participant for the Go/NoGo task across the four blocks where an emotional expression was set as the Go stimulus. The model for accuracy, measured by D-prime, in the Go/NoGo task was fitted to the data using normal skew family and including a random intercept for family and a population-level effect for group (COPMI vs. COPWMI). The model ran 40,000 Markov chain Monte Carlo (MCMC) iterations per chain across five chains. The model showed good convergence, indicated by R-hat values close to 1 for all parameters. Effective sample sizes (bulk-ESS and tail-ESS) were sufficiently high, indicating reliable parameter estimates. The model indicates an effect for being a COPMI on accuracy in the Go/NoGo task [*b* = −.197, 95% CI (−.293 to −.101)], namely, we observed a statistically significant decrease in Go/NoGo task accuracy of.197 units for COPMI compared to COPWMI. However, BF indicates that the evidence provided mildly favors the null hypothesis over the postulated alternative hypothesis (BF = .839).

We calculated mean reaction times per participant for the Go/NoGo task across the four blocks where an emotional expression was set as the Go stimulus. The model for reaction times in the Go/NoGo task was fitted to the data using normal skew family and including a random intercept for family and a population-level effect for group. The model ran 40,000 MCMC iterations per chain across five chains. The model showed good convergence, indicated by R-hat values close to 1 for all parameters. Effective sample sizes (bulk-ESS and tail-ESS) were sufficiently high, indicating reliable parameter estimates. The model indicates no effect of being a COPMI on reaction times in the Go/NoGo task [*b* = −3.876, 95% CI (−21.293 to 14.064)]. Furthermore, BF indicates that the evidence provided strongly favors the null hypothesis over the postulated alternative hypothesis (BF = .027).

#### 3.2.2 FER differences in the morphing task

Mean accuracy rates were computed for each participant across all emotions. The model for accuracy in the morphing task was fitted to the data using normal skew family and including a random intercept for family and a population-level effect for group. The model ran 40,000 MCMC iterations per chain across five chains. The model showed good convergence, indicated by R-hat values close to 1 for all parameters. Effective sample sizes (bulk-ESS and tail-ESS) were sufficiently high, indicating reliable parameter estimates. The model indicates a small effect of being COPMI on the accuracy in the morphing task [*b* = −.024, 95% CI (−.045 to −.003)], namely, we observed a statistically significant decrease in morphing task accuracy of.024 units for COPMI compared to COPWMI. However, BF indicates that the evidence provided strongly favors the null hypothesis over the alternative hypothesis (BF = .187). Subsequent models fit for accuracy for each emotion separately did not yield significant results (see **Table 4**).

**Table 4:** Models for accuracy per emotion in the morphing task.

	Standardized estimate	SD	95% CI_lower_	95% CI_upper_	BF
Joy accuracy	.000	.001	−.002	.002	.002
Anger accuracy	−.005	.006	−.018	.007	.012
Sadness accuracy	−.008	.011	−.031	.014	.012
Fear accuracy	−.010	.009	−.030	.008	.022

Mean reaction times were calculated for each participant across all trials and all emotions. The model for reaction times in the morphing task was fitted to the data using the Gaussian family and including a random intercept for family and a population-level effect for group. The model ran 40,000 MCMC iterations per chain across five chains. The model showed good convergence, indicated by R-hat values close to 1 for all parameters. Effective sample sizes (bulk-ESS and tail-ESS) were sufficiently high, indicating reliable parameter estimates. The model indicates no effect of being COPMI on reaction times in the morphing task [*b* = .095, 95% CI (−.228 to.417)]. BF indicates that the evidence provided strongly favors the null hypothesis over the alternative hypothesis (BF <.001).

#### 3.2.3 FER differences in the task presenting emotional video sequences

Mean accuracy was calculated for each participant across all video clips. The model for accuracy in the task presenting emotional video sequences was fitted to the data using normal skew family and including a random intercept for family and a population-level effect for group. The model ran 40,000 MCMC iterations per chain across five chains. The model showed good convergence, indicated by R-hat values close to 1 for all parameters. Effective sample sizes (bulk-ESS and tail-ESS) were sufficiently high, indicating reliable parameter estimates. The model indicates no effect of being COPMI on accuracy in a task presenting emotional video sequences [*b* = .001, 95% CI (−.011 to.014)]. BF indicates that the evidence provided strongly favors the null hypothesis over the alternative hypothesis (BF = .008). For a summary of the parameters of each Bayes model, see **Table 3**.

## 4 Discussion

Our study aimed to compare FER abilities between COPMI and those without (COPWMI), expanding beyond depression to various parental disorder types. We utilized diverse tasks involving static and dynamic stimuli, considering task influence on outcomes (27). Based on previous findings, we hypothesized that COPMI show FER impairments across all three tasks in accuracy as well as recognition speed in the two tasks, in which RT was measured. Contrasting our expectations, our results did not indicate a *general* FER deficit in COPMI compared to COPWMI. There was no effect of having a mentally ill parent on recognition speed (measured via reaction times) in either the Go/NoGo task or the morphing task. For accuracy, the models fit for the morphing task and the Go/NoGo task yielded a significant effect: COPMI were significantly less accurate in identifying emotional expressions in both tasks (see **Table 3**). However, the effect size was small and the Bayes factor calculated for the models was below 1, pointing toward the null hypothesis and suggesting that the effect could be due to noise or random variations. Furthermore, deconstructing the accuracy in the morphing task by fitting models for each emotion separately yielded no significant results (see **Table 4**).

In summary, our results suggest no effects of having a mentally ill parent on recognition speed and only little effects on accuracy for FER. This does not align with the majority of previous findings. Our divergent result could be based on numerous reasons, of which we consider three to be specifically interesting for future investigation: Firstly, our study exclusively includes parents with a mental disorder acute at the time of assessment, which differed from prior research, often including parents with a *history* of mental disorders rather than a current diagnosis (6, 21, 32, 42, 43). Research on the development of FER abilities and the influence of a parental mental illness on children suggests that this might make a relevant difference. Research on the influence of parental mental health found children to be especially sensitive to their parent’s mental health during the first years of life (39, 67–69).

In addition, FER abilities start to develop in early infancy (2, 4) and Pascalis et al. (70) argue for a sensitive period for face processing throughout the early years of life. Parental influence on children’s FER abilities is also strongest during infancy because parents are the main interaction partners for their children (12). Because mental illness is also associated with changes in facial expressivity (71–73), this means that COPMI are confronted with deviations in their main interaction partner’s facial expression during a sensitive period.

Taking into account the early development of FER and children being highly sensitive to parental mental health issues in infancy, it is possible that not the parental mental illness itself is associated with FER impairments in children, but rather the timing of the first parental disorder onset might be relevant. Therefore, it is possible that in our sample, we might not see an effect of having a mentally ill parent on FER abilities in COPMI, because the mental illness did not occur at a specific time point in the child’s life. Future research should take this into consideration by assessing FER in COPMI in the context of the timing of the parental mental disorder, optimally through prospective longitudinal studies.

Secondly, the COMPARE project was an at-risk evaluation, aiming to assess COPMI that do not show clinically relevant signs of a mental disorder themselves yet. It could therefore be due to selection bias that we included specifically those children that built up resilience from the parental mental disorder. In this case, those children would not show any FER impairments but would also not be representative of COPMI in general. We assessed internalizing and externalizing behaviors in COPMI, and while levels were significantly heightened in COPMI compared to COPWMI, mean *T*-scores were still below the clinical cutoff for COPMI, suggesting that they did not display clinically relevant levels of internalizing and/or externalizing behaviors. However, the cross-sectional study design did not allow any further insights, because we are unable to assess possible mental illness in the children’s future.

Lastly, past research mainly focused on children of parents with depression and mood disorders (6, 21, 32, 33, 37, 38, 43). To our knowledge, there is no study to date examining FER in a heterogeneous COPMI sample regarding parental disorder type. However, results found in children of parents with depression cannot necessarily be generalized to other disorders, because disorders have varying etiologies, symptom profiles, and neurobiological underpinnings. It is possible that FER is, in fact, not a transdiagnostic feature but rather only relevant for specific disorders and that the diversity in parental disorders in our sample might have diluted the potential FER effects for specific disorders. This could explain the trend for worse accuracy in COPMI compared to COPWMI in our sample. Future research should take a closer look at this when assessing FER impairments in COPMI by conducting subgroup analyses.

### 4.1 Strengths and limitations

While our study offers valuable insights, it is essential to acknowledge several limitations when interpreting our findings. The most significant limitation lies in the cross-sectional study design. While it allowed for comparing FER abilities between COPMI and COPWMI, it did not allow for prognostic conclusions regarding FER in COPMI. To be able to understand possible risk factors for transgenerational transmission of mental disorders and monitor their role in the onset and course of mental illness, longitudinal research is needed. Furthermore, our assessment paradigms also came with a set of limitations. The accuracy rates, particularly in the task presenting emotional video sequences, displayed strong ceiling effects, suggesting that the tasks may have been too easy for the children to depict a variability in FER abilities. Therefore, the task’s suitability for assessing FER impairments should be reconsidered for future studies, especially since we found a trend of COPMI being less accurate in FER in the other tasks. Future research should adapt this task further to mitigate ceiling effects. To address the issue of skewed data in our study, we employed Bayesian models with normal skew family (63), because they offer greater predictive utility compared to frequentist models using data transformations and, therefore, were the favorable solution in this context. Lastly, our study sample presents some limitations as well. While our sample is representative of the population assessing outpatient mental health services, sample sizes for parental disorder types are not homogeneous and, for some disorders, very small (e.g., *n* = 2 for feeding and eating disorders), preventing us from performing subgroup analyses to assess FER separately for different parental disorder types. Keeping these limitations in mind, the study still provides essential information for the field of FER in COPMI. Our study was the first to assess FER in a COPMI sample representative for outpatient mental health services. The diagnostic status of parents and children was assessed using state-of-the-art clinical interviews. The results were rated by trained clinicians, which assured proper diagnosis rather than self-report symptomatology. In addition, to our knowledge, this study was the first to assess FER in COPMI using three different tasks varying in their task demands for assessment.

### 4.2 Implications and conclusion

Our results suggest that FER impairments are not a transdiagnostic feature in COPMI. However, these results need to be confirmed by future research, especially including information on the time point of the onset of the parental disorder and taking a closer look at different disorders separately, for example by conducting subgroup analyses. Drawing practical implications from our study, our results suggest that COPMI are a heterogeneous group with many factors interplaying. Individual factors, such as parental disorder type and time point of disorder onset, need to be assessed and considered when developing COPMI interventions.

## Data availability statement

Data are not available due to ethical/legal/commercial restrictions. Requests to access the datasets should be directed to NW, naomi.werkmann@psychol.uni-giessen.de.

## Ethics statement

The studies involving humans were approved by Lokale Ethik-Kommission des Fachbereichs 06 (LEK-FB06), Justus-Liebig-Universität, Gießen, Germany. The studies were conducted in accordance with the local legislation and institutional requirements. Written informed consent for participation in this study was provided by the participants’ legal guardians/next of kin.

## Author contributions

NW: Methodology, Visualization, Writing – original draft, Writing – review & editing, Validation, Data curation, Formal analysis, Investigation. AL: Conceptualization, Investigation, Writing – review & editing. KH: Conceptualization, Data curation, Formal analysis, Investigation, Software, Writing – review & editing, Validation. RuS: Conceptualization, Funding acquisition, Project administration, Resources, Writing – review & editing. SW: Conceptualization, Funding acquisition, Project administration, Resources, Writing – review & editing. HC: Conceptualization, Funding acquisition, Project administration, Resources, Writing – review & editing. MK: Conceptualization, Formal analysis, Funding acquisition, Project administration, Resources, Software, Writing – review & editing. KO: Conceptualization, Funding acquisition, Project administration, Resources, Writing – review & editing. CR: Conceptualization, Funding acquisition, Project administration, Resources, Writing – review & editing. RiS: Conceptualization, Funding acquisition, Project administration, Resources, Writing – review & editing. LW: Conceptualization, Funding acquisition, Project administration, Resources, Writing – review & editing. A-LZ: Conceptualization, Funding acquisition, Project administration, Resources, Writing – review & editing. CS: Conceptualization, Funding acquisition, Project administration, Resources, Supervision, Writing – review & editing.

## The COMPARE-family research group

Study management: Stracke, Gilbert, Eitenmüller; biometry and data management: Awounvo, Kirchner, Klose; clinical study monitoring: Buntrock, Ebert; recruiting center Bielefeld: Schlarb; recruiting center Bochum: Margraf, Schneider, Friedrich, Teismann; recruiting center Gießen: Stark, Metzger; recruiting center Greifswald: Brakemeier, Wardenga, Hauck; recruiting center Landau: Glombiewski, Schröder, Heider; recruiting center Mainz: Jungmann, Witthöft; recruiting center Marburg: Rief, Eitenmüller.

## Supplementary material

Supplementary Table Bayesian Correlations between outcome measures and covariates including Person Correlation Coefficients and Credibility intervals.
