# Supplementary material for: Facial emotion recognition in children of parents with a mental illness
Source: Front Psychiatry. 2024 Jun 13;15:1366005. doi: 10.3389/fpsyt.2024.1366005 (PMC11210522; doi:10.3389/fpsyt.2024.1366005)
Supplement: Supplementary Table — Bayesian Correlations between outcome measures and covariates including Person Correlation Coefficients and Credibility intervals. [file Table_1.docx]

**Supplement**

**Table A.** *Bayesian Correlations between outcome measures and covariates including Person Correlation Coefficients and Credibility intervals*

|  |  | Accuracy Go/Nogo-Task | Accuracy MT | RT Go/Nogo Task | RT MT | RT MT Baseline | Accuracy MT Baseline | Accuracy VST | Gender | Age |
| --- | --- | --- | --- | --- | --- | --- | --- | --- | --- | --- |
| Accuracy MT | Pearson | **.413** |  |  |  |  |  |  |  |  |
|  | CI | **.287 - .521** |  |  |  |  |  |  |  |  |
| RT Go/Nogo Task | Pearson | **0.829** | **.222** |  |  |  |  |  |  |  |
|  | CI | **.779 - .869** | **.007 - .344** |  |  |  |  |  |  |  |
| RT MT | Pearson | **-.203** | .135 | **- .211** |  |  |  |  |  |  |
|  | CI | **-.338 - -.066** | -.007 - .268 | **-.334 - -.062** |  |  |  |  |  |  |
| RT MT Baseline | Pearson | -.131 | -.032 | -.097 | **.230** |  |  |  |  |  |
|  | CI | -.269 - .007 | -.166 - .115 | -.236 - .042 | **.095 - .365** |  |  |  |  |  |
| Accuracy MT Baseline | Pearson | **.195** | **.220** | .073 | .061 | **.192** |  |  |  |  |
|  | CI | **.055 - .325** | **.081 - .351** | -.074 - .207 | -.080 - .203 | **.048 - .319** |  |  |  |  |
| Accuracy VST | Pearson | **.259** | **.276** | **.145** | **-.144** | -.144 | .067 |  |  |  |
|  | CI | **.121 - .385** | **.140 - .403** | **.004 - .287** | **-.277 - -.001** | -.278 - .004 | -.069 - .207 |  |  |  |
| Gender | Pearson | .096 | **.206** | .086 | -.020 | .065 | **.208** | .118 |  |  |
|  | CI | -.052 - .232 | **.067 -.336** | -.054 - .224 | -.160 - .121 | -.079 - .204 | **.069 - .338** | -.021 - .253 |  |  |
| Age | Pearson | **.578** | **.290** | **.315** | -.031 | **-.231** | **.185** | **.234** | -.007 |  |
|  | BF | **.474 - .664** | **.153 - .413** | **.185 - .436** | -.173 - .103 | **-.360 - -.089** | **.041 - .316** | **.099 - .363** | -.144 - .133 |  |
| SES | Pearson | .024 | .091 | .073 | **-.159** | **-.169** | .056 | .092 | .037 | .013 |
|  | BF | -.119 - .166 | -.049 - .231 | -.065 - .214 | **-.287 - -.015** | **-.300 - -.028** | -.082 - .204 | -.042 - .234 | -.099 - .175 | -.123 - .157 |

MT = Morphing Task; RT = Reaction Times; VST = Task depicting emotional video sequences; CI = credibility interval, SES = socioeconomic status

Significant effects are printed in bold.
